# TRIM67 alleviates cerebral ischemia‒reperfusion injury by protecting neurons and inhibiting neuroinflammation via targeting IκBα for K63-linked polyubiquitination

**DOI:** 10.1186/s13578-023-01056-w

**Published:** 2023-05-29

**Authors:** Yongbo Yu, Qian Xia, Gaofeng Zhan, Shuai Gao, Tangrui Han, Meng Mao, Xing Li, Yonghong Wang

**Affiliations:** 1 Third Hospital of Shanxi Medical University, Shanxi Bethune Hospital, Shanxi Academy of Medical Sciences, Tongji Shanxi Hospital, Taiyuan, 030032 China; 2 Department of Anesthesiology, Hubei Key Laboratory of Geriatric Anesthesia and Perioperative Brain Health, and Wuhan Clinical Research Center for Geriatric Anesthesia, Tongji Hospital, Tongji Medical College, Huazhong University of Science and Technology, Wuhan, 430030 China; 3 Department of Neurosurgery, Shanxi Bethune Hospital, Shanxi Academy of Medical Sciences, Tongji Shanxi Hospital, Third Hospital of Shanxi Medical University, Taiyuan, 030032 China; 4 Department of Anesthesiology and Perioperative Medicine, Zhengzhou Central Hospital Affiliated to Zhengzhou University, Zhengzhou, 450007 China

**Keywords:** TRIM67, Cerebral ischemia‒reperfusion injury, Neuronal apoptosis, Neuroinflammation, IκBα, Ubiquitination

## Abstract

**Background:**

Excessive and unresolved neuroinflammation plays an important role in the pathophysiology of many neurological disorders, such as ischemic stroke, yet there are no effective treatments. Tripartite motif-containing 67 (TRIM67) plays a crucial role in the control of inflammatory disease and pathogen infection-induced inflammation; however, the role of TRIM67 in cerebral ischemia‒reperfusion injury remains poorly understood.

**Results:**

In the present study, we demonstrated that the expression level of TRIM67 was significantly reduced in middle cerebral artery occlusion and reperfusion (MCAO/R) mice and primary cultured microglia subjected to oxygen–glucose deprivation and reperfusion. Furthermore, a significant reduction in infarct size and neurological deficits was observed in mice after TRIM67 upregulation. Interestingly, TRIM67 upregulation alleviated neuroinflammation and cell death after cerebral ischemia‒reperfusion injury in MCAO/R mice. A mechanistic study showed that TRIM67 bound to IκBα, reduced K48-linked ubiquitination and increased K63-linked ubiquitination, thereby inhibiting its degradation and promoting the stability of IκBα, ultimately inhibiting NF-κB activity after cerebral ischemia.

**Conclusion:**

Taken together, this study demonstrated a previously unidentified mechanism whereby TRIM67 regulates neuroinflammation and neuronal apoptosis and strongly indicates that upregulation of TRIM67 may provide therapeutic benefits for ischemic stroke.

**Graphical Abstract:**

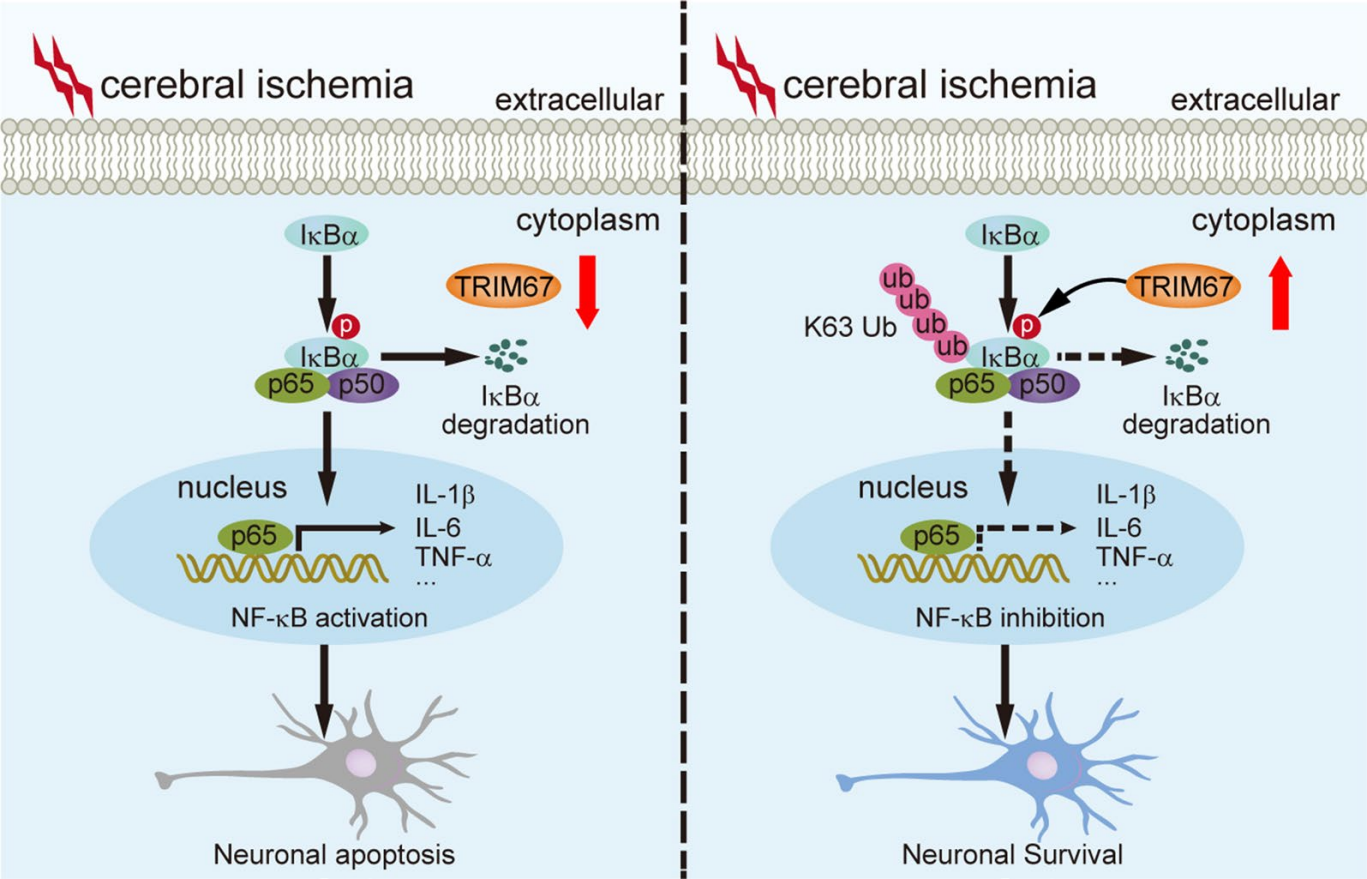

**Supplementary Information:**

The online version contains supplementary material available at 10.1186/s13578-023-01056-w.

## Introduction

Ischemic stroke is one of the major causes of mortality and disability worldwide [1]. Brain damage following cerebral ischemia involves complex pathogenic pathways [2, 3]. In the central nervous system (CNS), excessive release of proinflammatory cytokines or chemokines secondary to cerebral ischemia‒reperfusion injury can also cause irreversible neuronal damage, and unrestrained neuroinflammation can aggravate this damage [4, 5]. Accumulating evidence has shown that neuroinflammation plays a critical role in the pathogenesis of cerebral ischemia, and as such, has become a target for therapeutic intervention [6–8]. Due to an insufficient investigation of the pathogenesis of cerebral ischemia, therapeutic strategies to decrease neuroinflammation have not yielded the expected outcomes in clinical studies [9]. Accordingly, novel therapies capable of restraining ischemia-associated neuroinflammation could potentially prevent neuronal death and contribute to the treatment of ischemic stroke.

Nuclear factor kappa B (NF-κB), an essential early transcription regulator, is associated with various cellular events, especially apoptosis and inflammation [8, 10, 11]. Accumulating evidence has shown that ubiquitination, an important posttranslational modification, modulates the activation of NF-κB signaling pathways at various stages [12–15]. In the resting state, the NF-κB complex remains in an inactive form in the cytoplasm through inhibitor IκB proteins. Upon stimulation, the activity of IκB kinase (IKK) complexes, such as IKKα and IKKβ, is mediated by the K63-linked polyubiquitin chain. IKK complexes phosphorylate IκB proteins, which mediate K48-linked ubiquitination chains of IκB and target degradation by 26S-proteasome pathways [13, 16]. Following IκB degradation, NF-κB is released and transported to the nucleus and subsequently regulates the transcription of a series of genes, including inflammatory factors.

A previous study demonstrated that the tripartite motif-containing (TRIM) protein family, defined as having traditional ubiquitin E3 ligase activity, is emerging as a crucial regulator in numerous biological processes, such as inflammation, apoptosis, autophagy, cancer, and innate immunity [17–20]. TRIM proteins have a role in the regulation of NF-κB activation [21–25]. A previous study claimed that TRIM67 inhibits TNFα-triggered activation by competitively binding β-TrCP to IκBα [26]. Meanwhile, proteomic analysis found that TRIM67 expression dramatically decreases following stroke [27], implying that it might be involved in ischemic stroke. However, the significance and underlying mechanisms of NF-κB regulation of TRIM67 are not fully understood in ischemic stroke.

In this study, we observed that TRIM67 expression was significantly downregulated after cerebral ischemia‒reperfusion injury. Importantly, we found that TRIM67 overexpression significantly alleviated brain damage and cognitive deficits caused by cerebral ischemia‒reperfusion injury and decreased neuroinflammation and neuronal apoptosis. Mechanistically, we demonstrated that TRIM67 directly interacted with IκBα, reducing its K48-linked ubiquitination while increasing its K63-linked ubiquitination, thereby suppressing IκBα degradation and blocking NF-κB transcriptional activity. Together, these data showed a previously undefined role for TRIM67 in cerebral ischemia‒reperfusion injury and suggested that TRIM67/IκBα signaling might be a potential therapeutic target for ischemic stroke treatment.

## Materials and methods

### Experimental animals

C57BL/6 J mice (weighing 22–25 g) were obtained from Beijing River Laboratory Animal Corp. Ltd. Animals were raised in specific pathogen-free (SPF) conditions and kept in a 12-h light/dark cycle at a constant temperature of 24 °C. All animals were employed when they were 6–8 weeks old, and only male mice were used. All experiments were approved by the Experimental Animal Care and Use Committee of Tongji Hospital, Tongji Medical College, Huazhong University of Science and Technology (Wuhan, China) in accordance with the Animal Research: In vivo Reporting of Experiments (ARRIVE) guidelines [28]. The number of animals for each group was predetermined according to numbers reported in published studies or our prior experiment, and accurate animal numbers are given in the figure legends. Animals were randomized to experimental groups in all investigations.

### Transient focal cerebral ischemia

Left middle cerebral artery occlusion followed by reperfusion was conducted as we previously reported [29]. In brief, the mice were anesthetized with 2.5% isoflurane. A midline neck incision was used to expose the left external carotid artery (ECA), internal carotid artery (ICA), and common carotid artery (CCA). The ECA is ligated using two surgical 7–0 threads. One was toward the distal end of the ECA, while the other was near the proximal end, as close to the bifurcation of the ECA and ICA as feasible, followed by an incision between the ECA ligatures. Finally, a 2-cm long nylon filament with a diameter of 0.25 ± 0.03 mm was introduced into the ICA through the external carotid stump and progressed to occlude the origin of the middle cerebral artery (MCA). Cerebral blood flow (CBF) was monitored using a Laser Speckle Imaging System (RFLSI III, RWD Life Science, Shenzhen, China). The nylon filament was gently extracted from the ECA after an hour of ischemia for reperfusion. The incision at the neck was then closed. All of the animals were resuscitated on heat pads for two hours before being kept separately for additional investigation. The sham group mice underwent the same surgical operation technique as the control mice but without vaso-occlusive.

### Cell culture, transfection, oxygen and glucose deprivation/reperfusion (OGD/R)

As previously reported [30], primary cultured cortical neurons were generated from embryonic Day 17 mice. The separated cells were placed at a density of 1 × 10^7^ cells per well on 6-well culture plates. Briefly, cerebral cortices were gently separated, and the cell solution was filtered via a 70-m pore filter. Finally, the cells were cultured in vented T75 flasks (Corning, Shanghai, China) and incubated at 37 °C before being resuspended in high-glucose Dulbecco’s modified Eagle’s medium (DMEM, Thermo Fisher Scientific, Waltham, MA, USA) containing 10% fetal bovine serum (FBS, Gibco, Gaithersburg, MD, USA). Primary microglia were cultured according to our previously reported method [31]. After dissection for 10–14 days, primary microglial cells were extracted from the culture by shaking at 400 rpm for 6 h on a 37 °C rotary oscillator.

OGD/R was conducted as reported previously [32]. OGD/R was used as an in vitro model for mimicking cerebral ischemia‒reperfusion injury. Briefly, primary cultured neurons or microglia were maintained in preheated DMEM glucose-free medium (Gibco, Gaithersburg, USA) and incubated at 37 °C in a hypoxic incubator chamber with a 94% N_2_, 1% O_2_, and 5% CO_2_ gas combination. After one hour, the culture media was replaced with normal DMEM containing 10% heat-inactivated fetal bovine serum (Gibco), and the cells were returned to the standard humidified incubator chamber at 37 °C in a 5% CO_2_ environment, where reperfusion for 24 h began.

### Adeno-associated virus (AAV) viral vector transduction in mice

For in vivo viral infection experiments, the animals were infected with AAV2/6 viruses (2–3 × 10^12^ vg/ml) encoding CMV-GFP-Vector or CMV-GFP-TRIM67 by intracerebral injection, which induced TRIM67 overexpression. The AAV virus was injected using stereotaxic surgery. Four weeks before MCAO/R, the mice were sedated and injected with 500 nL of virus solution (50 nL/min) via glass electrodes linked to a stepper motor-driven microinjector into the hippocampal CA1 region, cerebral cortex, and striatum of the left hemisphere (Hamilton, Reno, USA). The following stereotaxic injection coordinates were used: hippocampal CA1 region (AP: − 2.00 mm, ML: − 1.55 mm, DV: − 1.55 mm), cerebral cortex region (AP: 0.00 mm, ML: − 2.05 mm, DV: − 1.50 mm), and striatum region (AP: 0.00 mm, ML: − 2.05 mm, DV: − 3.50 mm). To prevent reflux, the needle was left in place for an additional 5 min before being removed. The skin incision was then sutured, and all animals were resuscitated on thermal pads for two hours and then returned to the cage for further studies.

### 2,3,5-Triphenyl-2H-tetrazolium chloride (TTC) staining

Twenty-four hours after reperfusion, the mice were sacrificed. The brain was promptly and continuously retrieved and chopped into six 2-mm thick slices by a brain-cutting matrix (RWD). The slices were then submerged in 2% TTC (Sigma‒Aldrich, St. Louis, USA) solution in the dark for 15 min at 37 °C. Then, the slices were fixed in 4% PFA and photographed. ImageJ software was used to calculate the infarct volume. The volume of the infarct was determined as follows: (contralateral area-ipsilateral noninfarct area)/contralateral area × 100% = infarct size (%).

### Behavioral tests

Behavioral tests were conducted on eight animals in each group, with the exception of six animals in the sham group. Following surgery, animals were evaluated for neurological scores using the modified neurological severity score (mNSS), as previously reported [30]. In this study, we conducted the Morris water maze (MWM) test to detect spatial learning and memory, and the novel object recognition (NOR) task was performed to examine hippocampal-dependent memory as reported previously [33, 34].

### Western blotting analysis

After reperfusion ranging from 12 to 72 h, radioimmune-precipitation assay (RIPA) buffer (Beyotime Biotechnology, Shanghai, China) was used to produce protein lysates from mouse peri-infarct cortex, hippocampus and striatum brain tissues and to treat primary cultured neurons (Thermo, Waltham, MA, USA). Lysates were centrifuged for 15 min at 4 °C at 14,000*g*. Supernatants were combined with an equivalent volume of 2 × loading buffer (Beyotime Biotechnology) and heated for 10 min until denaturation. SDS‒PAGE was utilized to separate the protein lysates, which were then transferred to PVDF membranes. Overnight at 4 °C, the membranes were treated with primary antibodies. The antibodies utilized in this study are listed in Additional file 4: Table S1. After three washes with TBST buffer, proteins were identified by incubating the membranes for 60 min at room temperature with horseradish peroxidase-conjugated secondary antibodies (Cell Signaling Technology, Danvers, USA) and an enhanced chemiluminescence substrate kit (Thermo Pierce, Rockford, USA), and ImageJ software was used to calculate band intensities.

### Co-immunoprecipitation (co-IP)

For co-immunoprecipitation, cells were lysed in immunoprecipitation (IP) buffer (Beyotime Biotechnology) supplemented with cOmplete™ protease inhibitor cocktail tablets (5 mg/ml; Roche, Basel, Switzerland) at 4 °C for 15 min. The extract was then centrifuged at 14,000 ×*g* for an additional 15 min, and the supernatant was gathered and employed. Anti-Myc or -Flag antibodies were added to suitable cell lysates, followed by incubation for 12 h using a shaker at 4 °C. After that, cell lysates with antibodies were incubated for 4 h at 4 °C with protein A/G plus agarose (Santa Cruz) and washed three times with precooled PBS buffer. Then, 2 × SDS–PAGE loading buffer was added to the samples followed by boiling at 95 °C for 5 min, and immunoblot analysis was performed to detect the protein expression.

### Enzyme-linked immunosorbent assay (ELISA)

Detection of IL-1β, IL-6, TNF-α, CXCL1, and CCL2 in the tissues was determined with a mouse IL-1β, IL-6, TNF-α, CXCL1, and CCL2 enzyme-linked immunosorbent assay (ELISA) kit (R&D Systems, Wiesbaden, Germany), and the results were expressed as picograms per milliliter.

### Real-time quantitative PCR (RT-qPCR)

As previously reported [35], RT-qPCR was used. Using TRIzol reagent, total RNA was extracted from brain tissues (Invitrogen, Carlsbad, USA). To obtain cDNA, the PrimeScript^®^ RT Reagent Kit (Takara, Kyoto, Japan) was utilized. RT-qPCR was then performed using a StepOnePlus™ Real-Time PCR System (Applied Biosystems, Foster City, USA) with a Light Cycler 96 (Roche) and SYBR Green PCR MIX (Takara). Primer Express software version 1.5 (Applied Biosystems) was used to construct the primers, and the sequences are presented in Additional file 5: Table S2. The 2^−ΔΔCt^ technique was used to analyze gene expression, and the relative gene expression levels were adjusted to *β-actin*.

### Immunofluorescence staining

Immunofluorescence staining was performed in accordance with a previously reported methodology [31]. Following heat-mediated antigen retrieval, the tissue slices were blocked with donkey serum for 60 min at 37 °C, followed by an overnight incubation with the primary antibodies at 4 °C. After three washes with PBS, all sections were incubated for four hours at room temperature with FITC-conjugated secondary antibody. After washing the slides three times with PBS, all sections were counterstained for 5 min at room temperature with 4′,6-diamidino-2-phenylindole (DAPI, US Everbright Inc.). A fluorescence microscope was used to evaluate all photographs (Olympus BX53).

### TUNEL staining

The immunofluorescent terminal deoxynucleotidyl transferase dUTP nick-end labeling (TUNEL) experiment was performed as previously described using the In Situ Cell Death Detection Kit (Roche) [33]. A fluorescence microscope was used to evaluate all photographs (Olympus BX53). The cell death percentage was estimated by dividing the total number of TUNEL-positive nuclei (green) by the total number of DAPI-positive nuclei (blue). Images were taken in the ischemia area and analyzed quantitatively with ImageJ software.

### Statistical analysis

GraphPad Prism 8.0 was used to analyze the data (GraphPad Software Inc., San Diego, CA, USA). Furthermore, all data are expressed as the mean ± SD from at least three independent experiments. Multiple group comparisons (> 2 groups) were performed using one-way ANOVA with Tukey’s post hoc test. The unpaired two-tailed Student's t test was used to examine differences between two groups. The training phase (6 days) of the MWM test was examined using repeated-measures ANOVA. The discontinuous data of neurological deficit score and times of the animals crossing over the platform location during the probe trial on Day 7 were analyzed by the Kruskal‒Wallis nonparametric test. A statistically significant difference between experimental outcomes was defined as a *p* value less than 0.05.

## Results

### TRIM67 was downregulated after experimental ischemic stroke

**Fig. 1:**
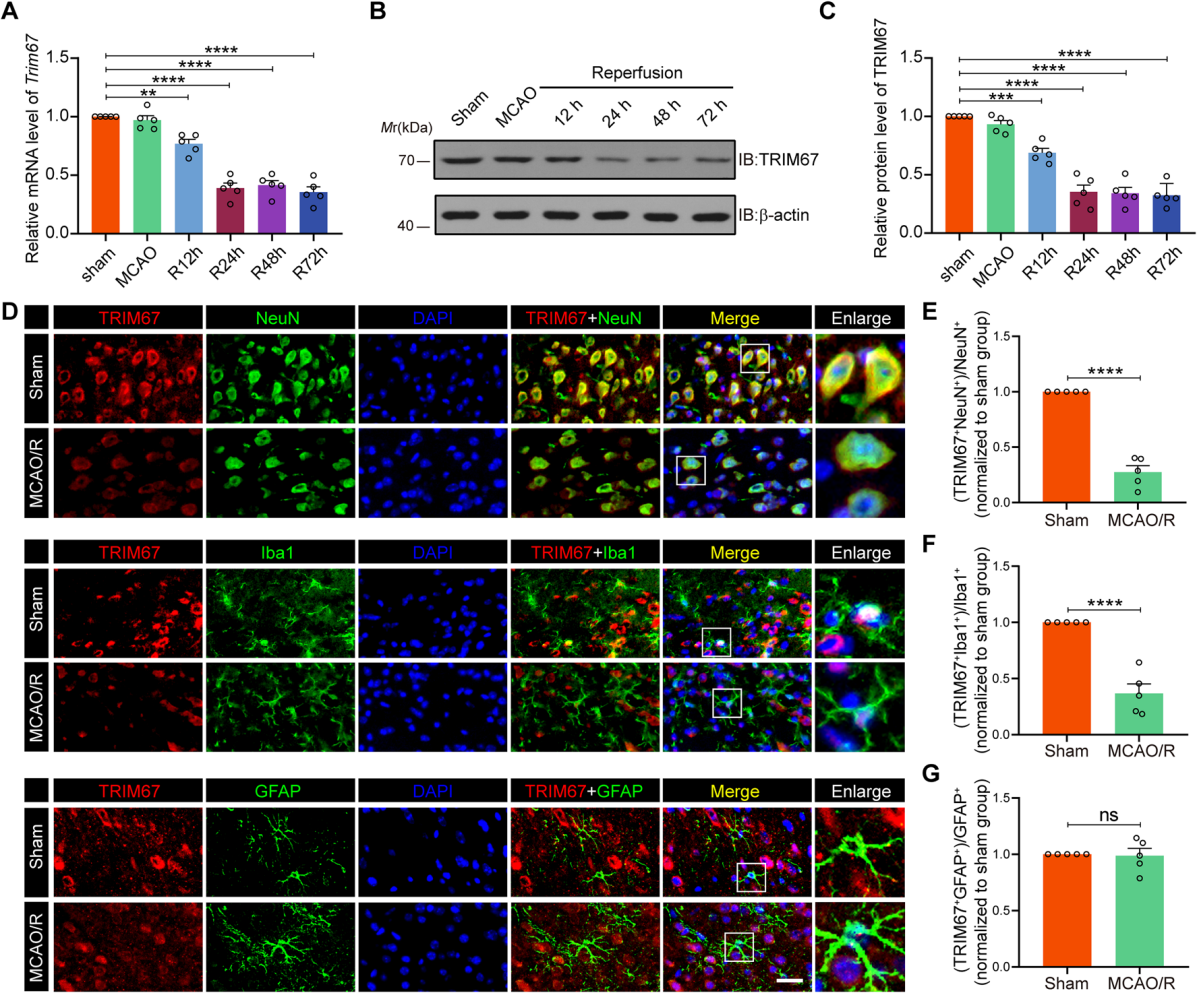
The expression of TRIM67 decreased after cerebral ischemia‒reperfusion injury. **A** and **B** RT-qPCR (**A**) and western blotting (**B**) assays showing *Trim67* mRNA and protein expression in mouse peri-infarct cortex, hippocampus and striatum tissues at the specified periods following middle cerebral artery occlusion/reperfusion (MCAO/R) (n = 5). **C** Quantification of TRIM67 expression normalized to that of β-actin in **B**. **D** Representative double immunostaining of TRIM67 (red) with NeuN (a neuronal marker, green), Iba-1 (a microglial marker, green) and GFAP (an astrocyte glial marker, green) from ischemic penumbra of brain tissue after MCAO surgery. **E**–**G** Quantification of TRIM67 fluorescence intensity in NeuN-, Iba1-, and GFAP-positive cells were quantified using ImageJ. The analytical graph was presented as normalized to the sham group. Scale bars, 40 μm. (n = 5). The mean ± SD are displayed for all data. **p < 0.01, ***p < 0.001 and ****p < 0.0001

To explore the function of TRIM67 in cerebral ischemia‒reperfusion injury, we first detected whether TRIM67 expression was altered in vivo and in vitro. First, an experimental stroke model was developed by performing 1 h of MCAO surgery followed by varied intervals of reperfusion ranging from 12 to 72 h. The mouse peri-infarct hippocampus, cortex and striatum were extracted, and RT-qPCR analysis revealed that the level of *Trim67* mRNA showed a continuous decrease and reached a plateau over 24 h after cerebral ischemia‒reperfusion injury (Fig. 1A). Western blotting showed similar changes in expression at the protein level (Fig. 1B, C). Furthermore, we used immunofluorescence staining to examine the protein level of TRIM67 in the cortex of various experimental groups. The results revealed that TRIM67 were primarily expressed in neurons and microglia to a lesser extent in astrocytes (Fig. 1D). Moreover, these results were consistent with the RT-qPCR and immunoblots results, showing that TRIM67 fluorescence intensity in neurons and microglia was less intense in the MCAO/R-operated animals (Fig. 1E–G). Then, the OGD/R model was then developed to replicate ischemia‒reperfusion injury in vitro. Western blotting and RT-qPCR were performed to assess the protein and mRNA levels of TRIM67 in primary cultured neurons and microglia. The results showed that the level of TRIM67 dramatically decreased after 1 h of OGD treatment and 24 h of reoxygenation in primary neurons and microglia (Additional file 1: Fig. S1 and Additional file 2: Fig. S2), which was consistent with the in vivo experimental findings. Collectively, these results indicate that the expression level of TRIM67 was reduced after cerebral ischemia‒reperfusion injury.

### TRIM67 upregulation reduced the neurofunctional deficits of mice after MCAO/R

**Fig. 2:**
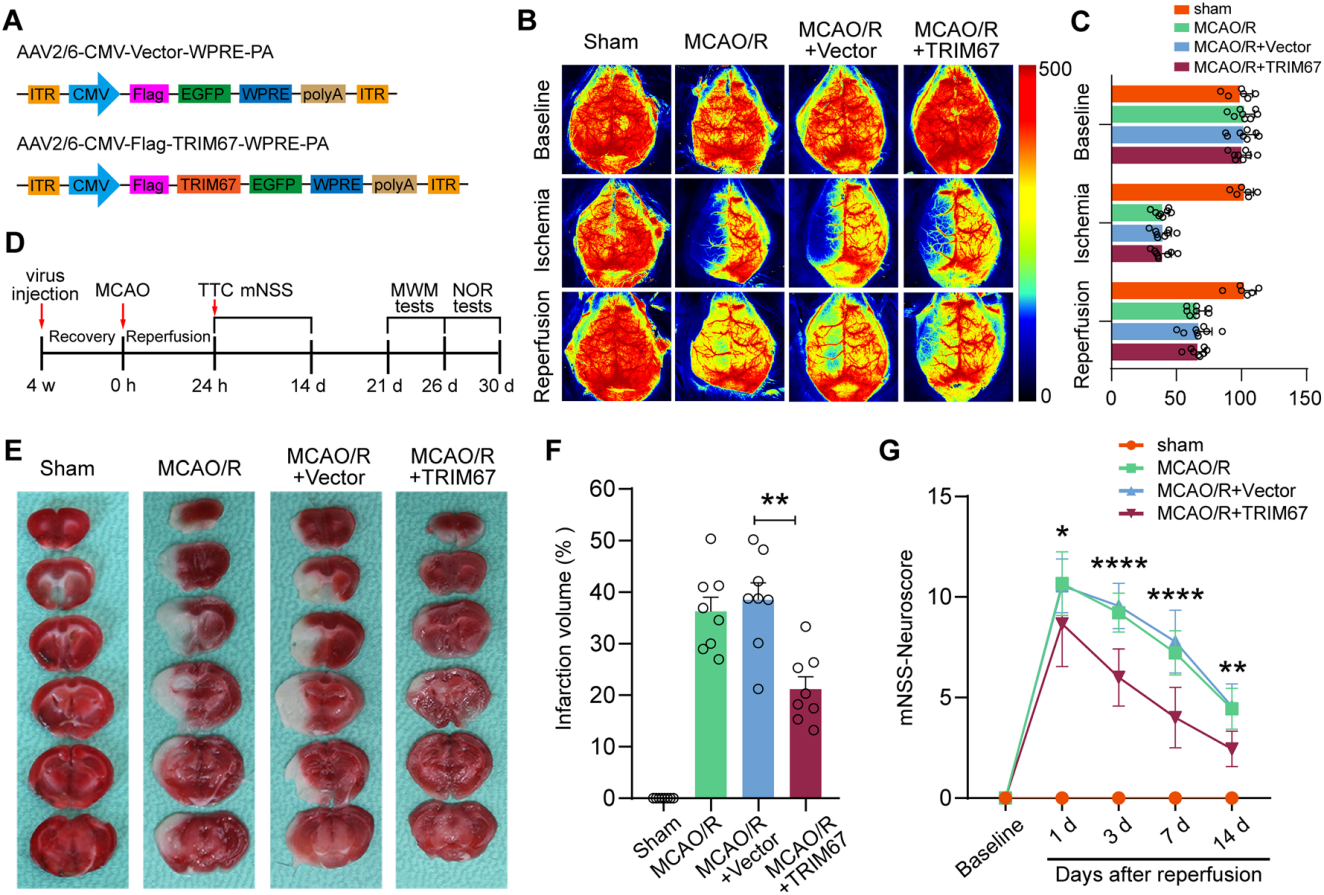
TRIM67 upregulation reduces the infarct volume and improves neurological function in mice after cerebral ischemia‒reperfusion injury. **A** Schematic of CMV-dependent adeno-associated virus for vectors or wild-type TRIM67 overexpression. **B** Cerebral blood flow monitored using 2-dimensional laser speckle imaging techniques before, during MCAO, and reperfusion. **C** The results are expressed as the percent change from baseline (pre-MCAO). **D** Diagram showing the experimental process. **E** After 24 h of MCAO, representative brain slices were stained with TTC. **F** Infarct volume quantitative analysis (n = 8). (G) The modified neurological severity score (mNSS) was used to determine the neurological deficiency score (n = 8). The mean ± SD are displayed for all data. *p < 0.05, **p < 0.01, ***p < 0.001 and ****p < 0.0001

Based on the findings of the in vivo and in vitro tests, we next studied whether TRIM67 upregulation could protect mice against cerebral ischemia‒reperfusion injury in vivo. For this purpose, we employed an AAV2/6 bearing the TRIM67 coding sequence to upregulate TRIM67 in mouse brains. As an experimental control, the same EGFP-expressing adeno-associated viral vector was used (Fig. 2A). Adult C57/BL6 male mice were stereotactically injected with the viruses into the hippocampal CA1 area, cerebral cortex, and striatum. Four weeks following AAV injection, mice were subjected to MCAO surgery for 1 h, followed by reperfusion. The AAV-Vector- and AAV-TRIM67-treated mice exhibited comparable regional CBF during MCAO and reperfusion (Fig. 2B, C). Subsequently, we conducted histological and behavioral studies at the designated time periods following reperfusion (Fig. 2D). First, AAV-TRIM67 infection efficiency in brain tissue was validated by western blotting and RT-qPCR, revealing that both the mRNA and protein levels of TRIM67 were enhanced in mice treated with AAV-TRIM67 compared to vector-treated animals (Additional file 3: Fig. S3). Next, the infarct size was examined by TTC staining, which revealed a notable decrease in infarct volume in TRIM67-overexpressing mice compared to the vector control animals (Fig. 2E, F). Next, neurological deficits were assessed based on a modified neurological severity score (mNSS). The results indicated a substantial genotypic difference, with TRIM67-overexpressing animals scoring better than vector-treated mice (Fig. 2G). These results collectively indicated that TRIM67 upregulation ameliorated cerebral ischemia‒reperfusion injury.

### TRIM67 alleviates cognitive impairment induced by cerebral ischemia‒reperfusion injury in mice

**Fig. 3:**
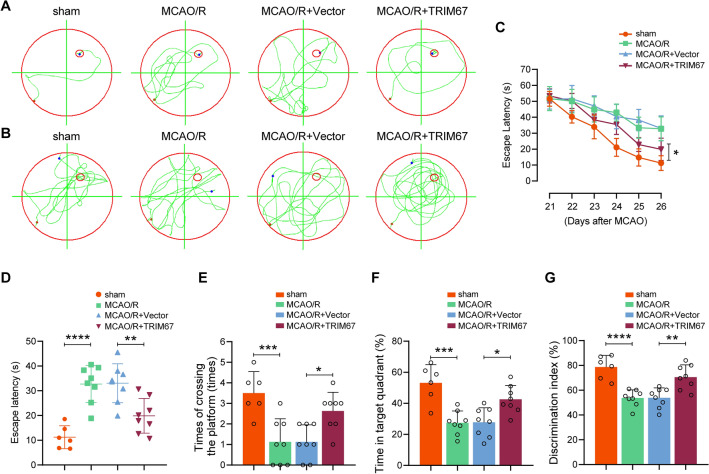
TRIM67 upregulation preserves cognitive function following cerebral ischemia‒reperfusion injury in mice. **A** and **B** Morris water maze (MWM) swimming traces of mice in latency and probe trials. **C** and **D** In the MWM tests, escape latency to uncover the hidden platform in the latency trial. **E** The number of times the mice passed the target platform position on Day 7 during the probe trials. **F** Time spent in the target quadrant in the probe trial on Day 7. **G** Preference index for the novel object in the novel object recognition (NOR) task. The discrimination index was recorded in the test phase. n = 6–8 mice per group. The mean ± SD are displayed for all data. *p < 0.05, **p < 0.01, ***p < 0.001 and ****p < 0.0001

We then examined the impact of TRIM67 on cognitive impairments following cerebral ischemia‒reperfusion injury. We first performed the MWM test to measure mouse hippocampus-dependent spatial learning and memory function. Representative swimming tracings of the mice during training and probing trials are presented in Fig. 3A, B. Under ischemic stroke, the search tracings of the TRIM67-overexpressing mice were more concentrated in the target or surrounding quadrants, whereas the vector groups were more random (Fig. 3B). The results showed that the mice treated with TRIM67 upregulation took less time to reach the hidden platform (Fig. 3C, D). During the probe trials on Day 7, the TRIM67-upregulated mice consistently exhibited more platform crossings (Fig. 3E) and spent more time in the targeted quadrant (Fig. 3F). These results collectively indicated TRIM67-overexpressing mice displayed remarkable cognitive improvement. Next, we detected mouse memory in the NOR task. The MCAO/R + AAV-TRIM67 group showed declarative recognition memory improvement compared to the MCAO/R + Vector group, as indicated by a greater preference for the novel object (Fig. 3G). Overall, these results suggest that TRIM67 upregulation ameliorated the cognitive function injury induced by cerebral ischemia‒reperfusion injury.

### TRIM67 exerts a neuroprotective effect against cerebral ischemia‒reperfusion injury by suppressing cell apoptosis

**Fig. 4:**
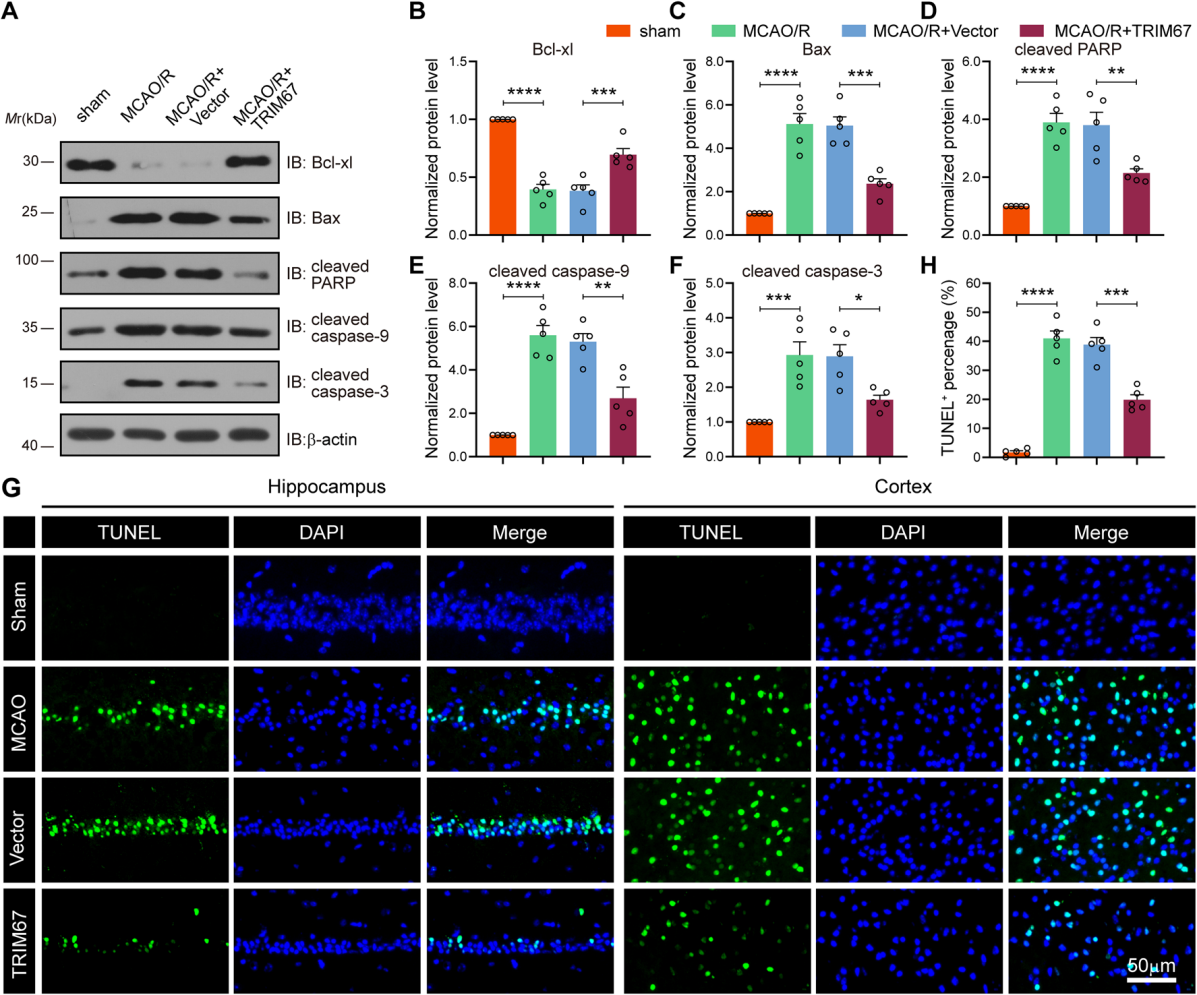
TRIM67 upregulation decreases cell apoptosis in mice after cerebral ischemia‒reperfusion injury. **A** The expression of Bcl-xl, Bax, cleaved caspase-3, -9, and cleaved PARP in vivo was examined by western blotting. **B**–**F** Quantitative analysis of apoptosis-related chemicals is shown in **A** (n = 5). **G** TUNEL test demonstrating cell death in the MCAO/R mouse peri-infarct hippocampus and cortex. Scale bar: 50 μm. **H** TUNEL-positive cells after MCAO/R were counted (n = 5). The mean ± SD are displayed for all data. *p < 0.05, **p < 0.01, ***p < 0.001 and ****p < 0.0001

Our in vivo studies suggested that TRIM67 upregulation improved neurofunction and cognitive function against cerebral ischemia‒reperfusion injury. Therefore, we hypothesized that TRIM67 exerted neuroprotection by an anti-apoptosis pathway in vivo. Four weeks following AAV injection, mice were treated 1 h after MCAO surgery, accompanied by reperfusion. We first investigated the impact of TRIM67 on the production of apoptotic proteins such as anti-apoptotic Bcl-xl, pro-apoptotic Bax (essential apoptotic regulators of mitochondria), and the cleaved form of caspase-3, -9, and PARP following ischemic injury. Western blotting assays revealed that MCAO/R dramatically decreased the protein level of anti-apoptotic Bcl-xl while increasing the expression of pro-apoptotic Bax and the cleaved form of caspase-3, -9, and PARP. However, TRIM67 overexpression reversed this tendency, as seen by the reduced expression of these proteins (Fig. 4A–F). Meanwhile, cell apoptosis caused by cerebral ischemia‒reperfusion injury was examined using TUNEL staining. The results demonstrated that TRIM67 overexpression successfully relieved the apoptosis produced by MCAO/R in the cortex and hippocampus, as evidenced by a reduction in TUNEL-positive cells (Fig. 4G, H). These results demonstrated the antiapoptotic effects of TRIM67 in cerebral ischemia‒reperfusion injury.

### TRIM67 alleviates neuroinflammation after cerebral ischemia‒reperfusion injury

**Fig. 5:**
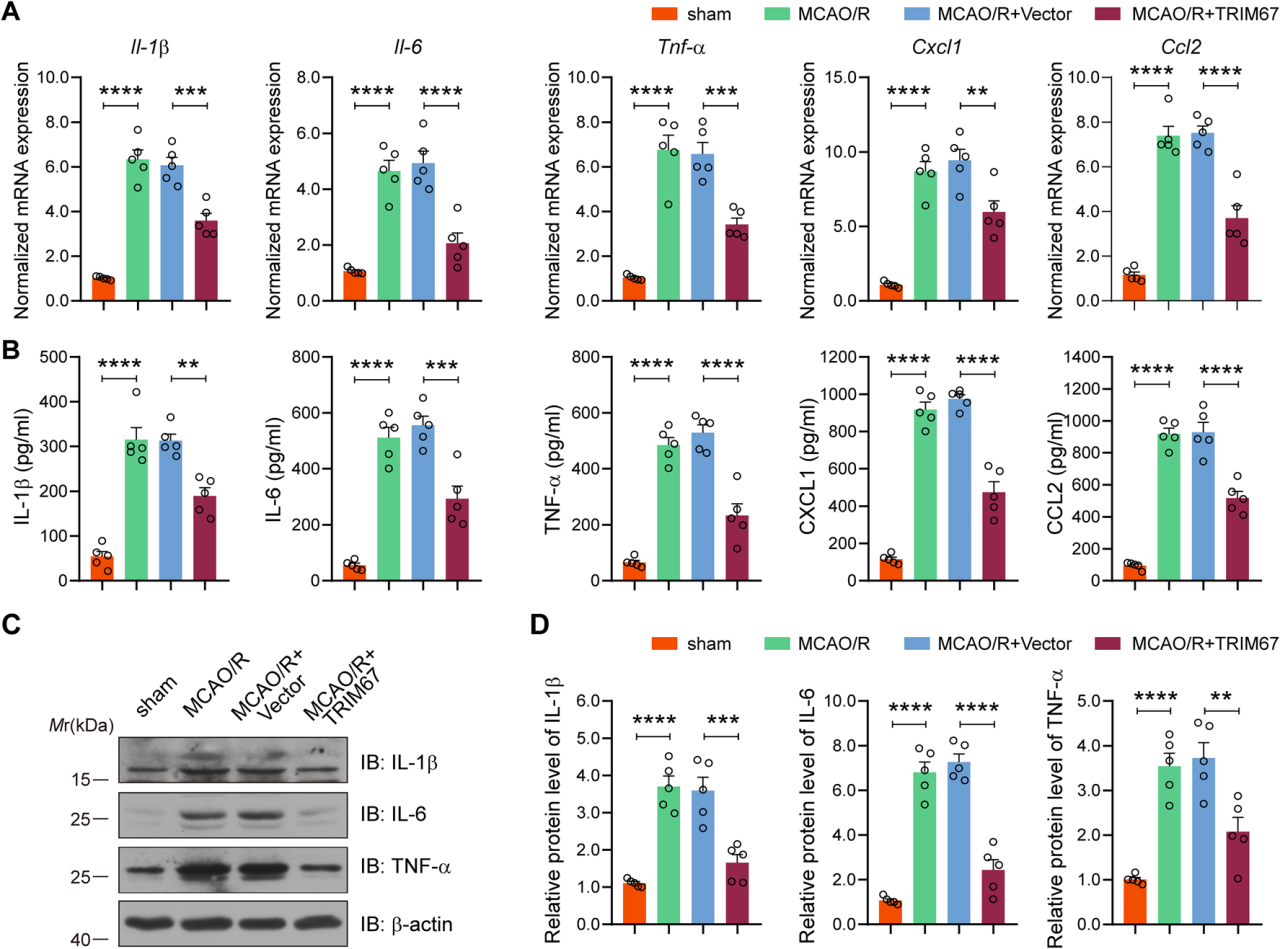
TRIM67 upregulation reduces postischemic neuroinflammation. RT-qPCR and ELISA were used to determine the mRNA (**A**) and protein (**B**) expression of IL-1β, IL-6, TNF-α, CXCL1, and CCL2 in peri-infarct cortex, hippocampus and striatum brain tissues (n = 5). **C** western blotting investigation of IL-1β, IL-6, and TNF-α expression in peri-infarct cortex, hippocampus and striatum brain tissues. **D** Quantitative analysis of inflammatory cytokines is shown in **C** (n = 5). The mean ± SD are displayed for all data. **p < 0.01, ***p < 0.001 and ****p < 0.0001

Cerebral ischemia‒reperfusion injury can cause an inflammatory reaction in the brain, and excessive inflammation is associated with neurotoxic effects on ischemic neuronal death. Thus, we next evaluated the effects of TRIM67 on inflammatory responses in mouse brain tissue at 24 h after reperfusion, and we measured the mRNA and protein levels of inflammatory cytokines and chemokines, including IL-1β, IL-6, TNF-α, CXCL1, and CCL2. RT-qPCR analysis revealed that cerebral ischemia‒reperfusion injury dramatically increased the mRNA levels of proinflammatory cytokines and chemokines, which were inhibited by TRIM67 upregulation (Fig. 5A). We also used ELISA to validate the protein levels of IL-1β, IL-6, TNF-α, CXCL1, and CCL2. The findings showed that the aforementioned molecules were elevated during MCAO/R but were greatly inhibited by TRIM67 upregulation (Fig. 5B). Meanwhile, western blotting was used to evaluate the protein abundance of pro-inflammatory markers (iNOS and CD16/32). The results indicated that iNOS and CD16/32 levels were dramatically upregulated, which was inhibited by TRIM67 overexpression (Fig. 5C, D). Overall, our data indicate that TRIM67 decreases inflammatory markers in brain tissue following cerebral ischemia‒reperfusion injury.

### TRIM67 functions as a negative regulator of the NF-κB signaling pathway

**Fig. 6:**
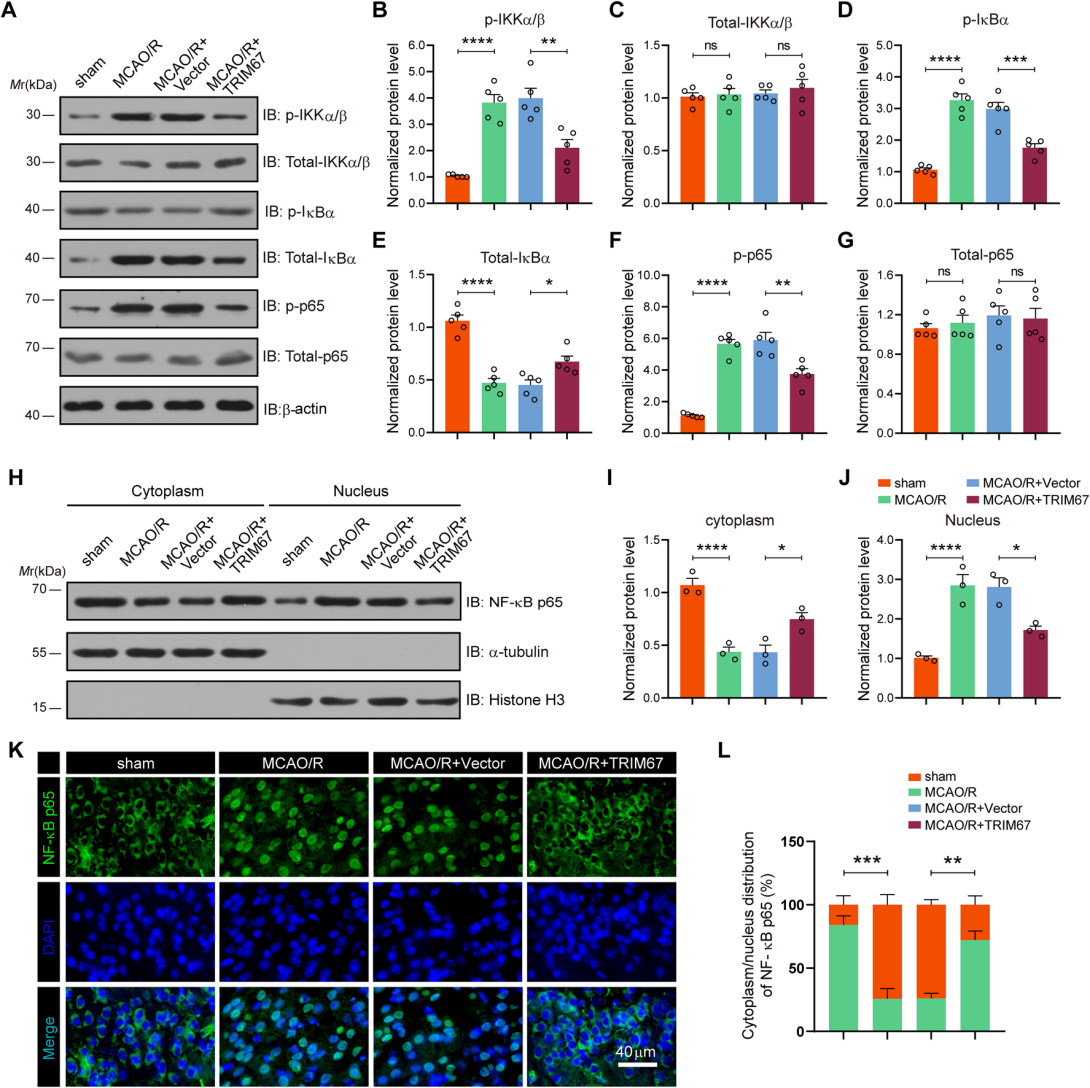
TRIM67 suppressed the activation of the NF-κB signaling pathway induced by cerebral ischemia‒reperfusion injury. **A** In vivo study of essential kinases in the NF-κB pathway using western blotting. **B**–**G** Quantitative analysis of p-IKKα/β, total-IKKα/β, p-IκBα, total-IκBα, p-p65 and total-p65 are shown in **A** (n = 5). **H** In vivo western blotting investigation of NF-κB p65 levels in the cytoplasm and nucleus. **I** and **J** NF-κB p65 quantitative analysis is displayed in (H) (n = 3). **K** NF-κB p65 expression in mice exposed to MCAO/R as seen in immunofluorescence images, DAPI (blue)/NF-κB p65 (green). Scale bar: 40 μm. **L** The levels of NF-κB p65 in the cytoplasm and nucleus were quantified. The mean ± SD are displayed for all data. *p < 0.05, **p < 0.01, ***p < 0.001 and ****p < 0.0001

Emerging evidence suggests that the inflammatory signaling pathway is an essential modulator of neuroinflammatory processes in various CNS diseases. Because the NF-κB pathway plays a critical role in neuroinflammation and apoptosis, we evaluated the impact of TRIM67 upregulation on NF-κB signaling activation. For this purpose, we conducted western blotting to detect total protein and phosphorylated levels of the key kinases involved in NF-κB signaling (Fig. 6A–G). The results demonstrated that cerebral ischemia‒reperfusion injury greatly increased the expression levels of phosphorylated IKKα/β (Ser176/180), IκBα (Ser32/36), and p65 (Ser536), which was considerably reversed by TRIM67 overexpression. As a result, NF-κB activation is characterized by an increase in NF-κB p65 nuclear transport. We investigated the subcellular localization of NF-κB p65 by western blotting (Fig. 6H–J). The findings suggested that, compared to MCAO/R alone, TRIM67 overexpression significantly decreased the protein levels of NF-κB p65 in the nucleus. Additionally, immunofluorescence analysis further confirmed these outcomes (Fig. 6K, L). These results indicated that TRIM67 upregulation prevented NF-κB signaling pathway activation in mice subjected to cerebral ischemia‒reperfusion injury.

### TRIM67 enhanced IκBα protein stability by increasing K63-linked ubiquitination of IκBα

**Fig. 7:**
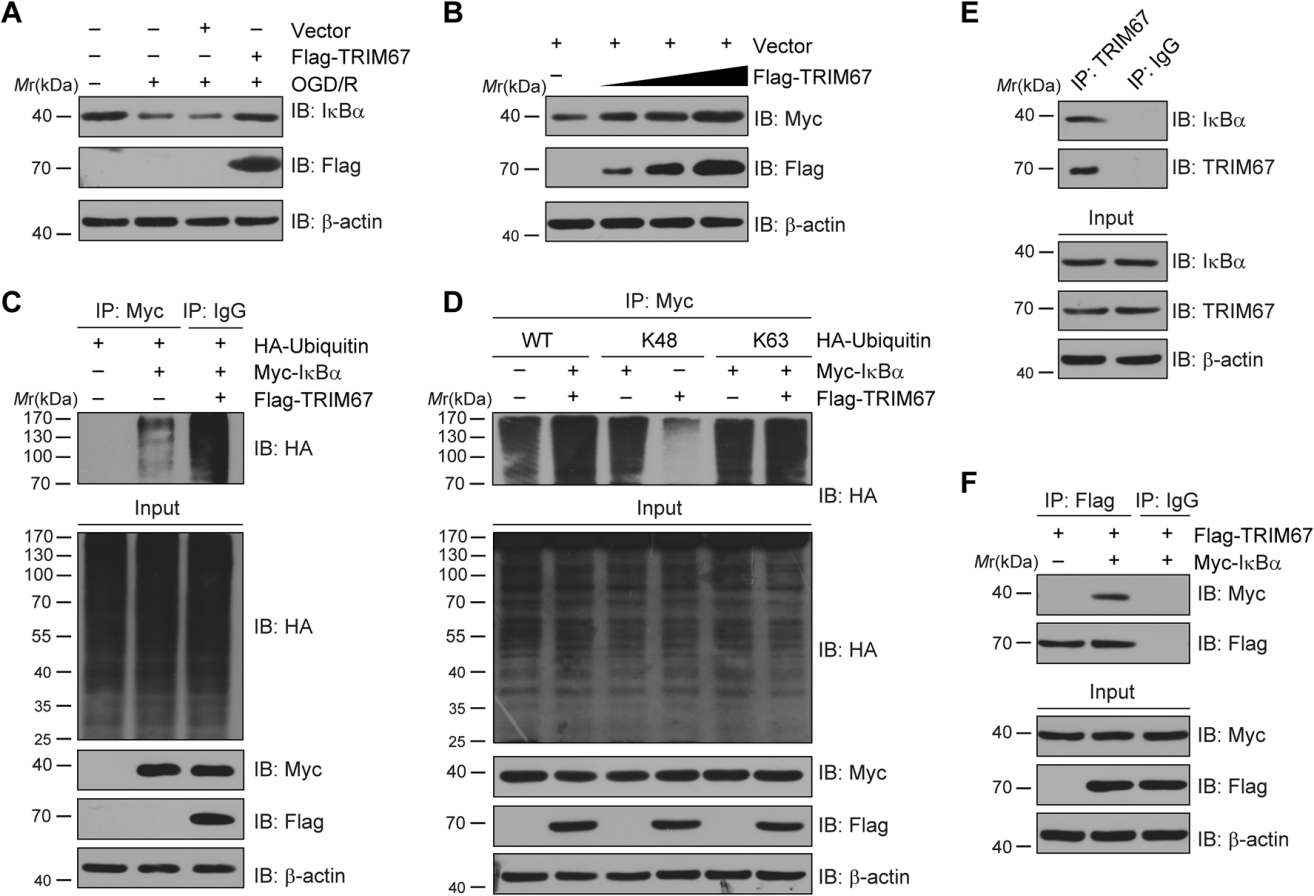
TRIM67 inhibits NF-κB signaling by regulating the level of K48- and K63-linked ubiquitination of IκBα. **A** Primary cultured microglia were infected with recombinant adenovirus carrying Flag-TRIM67 and treated with OGD/R. Western blotting was used to assess the effect of TRIM67 on total IκBα protein levels. **B** In HEK293T cells transfected with Flag-TRIM67 and Myc-IκBα, the effects of TRIM67 overexpression on IκBα degradation were studied. **C** In HEK293T cells transfected with Flag-TRIM67, Myc-IκBα, and HA-ubiquitin, a representative co-IP study was performed to identify the ubiquitination of IκBα. **D** In HEK293T cells transfected with Flag-TRIM67, Myc-IκBα, and Flag-K48/K63-Ubiquitin (Ub), a representative co-IP analysis was performed to assess the degree of K48- and K63-linked polyubiquitination of IκBα. **E** A representative co-IP study was performed to examine the interaction of endogenous TRIM67 with IκBα in brain tissue. **F** A representative co-IP study was performed to examine the interaction of ectopically expressed TRIM67 with IκBα in HEK293T cells transfected with Flag-TRIM67 and Myc-IκBα plasmids. Data are representative of three independent experiments

The precise mechanism by which TRIM67 decreased ischemia‒reperfusion injury-mediated NF-κB activity was then investigated. A previous study claimed that TRIM67 inhibits the β-TrCP-mediated degradation of IκBα, a negative regulator of NF-κB signaling. In our study, as shown in Fig. 6A, the total protein levels of IKKα/β and p65 remained unchanged, except for IκBα, which rose after TRIM67 overexpression. The results were further validated in primary cultured microglial cells challenged with OGD/R, as shown in Fig. 7A. TRIM67 is a RING domain E3 ubiquitin ligase that mediates protein ubiquitination, which regulates protein stability. Hence, we hypothesized that TRIM67 is involved in promoting IκBα protein stability. We discovered that increasing the dose of Flag-tagged TRIM67 contributed to significantly higher Myc-tagged IκBα protein levels (Fig. 7B). Next, we tested the effect of TRIM67 on the ubiquitination level of IκBα. Co‑IP indicated that TRIM67 promoted the ubiquitin modification of IκBα (Fig. 7C). In addition, Flag-tagged TRIM67 and Myc-tagged IκBα plasmids were cotransfected with HA-tagged wild-type ubiquitin, lysine 63 (K63)-only ubiquitin, or lysine 48 (K48)-only ubiquitin plasmids in HEK293T cells. The results demonstrated that TRIM67 overexpression inhibited K48-linked ubiquitination of IκBα while increasing K63-linked ubiquitination of IκBα (Fig. 7D). Polyubiquitin chain production via K48 of ubiquitin facilitates modified protein degradation; however, K63-linked polyubiquitin chains generally do not lead to substrate degradation but rather mediate low-affinity binding of other proteins. To explore whether TRIM67 directly binds to IκBα, co-IP assays were performed using lysates prepared from brain tissue. The results demonstrated that the two proteins could bind with each other (Fig. 7E). Meanwhile, HEK293T cells were transiently transduced with Flag-tagged TRIM67 and Myc-tagged IκBα expression vectors. Co‑IP showed that TRIM67 interacted with IκBα (Fig. 7F). Taken together, these findings revealed that TRIM67 enhanced IκBα protein stability by facilitating K63-linked ubiquitination of IκBα and inhibiting K48-linked ubiquitination of IκBα, causing the loss of proteasomal-mediated protein degradation.

## Discussion

Worldwide, ischemic stroke is a leading cause of death. Although knowledge of cerebral ischemia pathophysiology has advanced and new classes of drugs have been discovered, stroke remains an exclusive disease state [36–38]. Therefore, treating cerebral ischemia-induced damage necessitates prompt and effective management. An in vivo transient focal cerebral ischemia‒reperfusion injury model and in vitro OGD/R-induced damage models were utilized in this study to determine the effects of TRIM67 as a neuroprotective factor against ischemic stroke. Reducing the severity of cognitive impairments by effectively resolving neuroinflammation and neuronal death throughout the course of stroke may be possible. Here, a reduction in TRIM67 expression was observed in both animal and cell experiments. Importantly, TRIM67 interacted with IκBα, thus potentiating its K63-linked ubiquitination but reducing its K48-linked ubiquitination, enhancing the stability of IκBα protein, thereby suppressing the activity of the NF-κB signaling pathways and resulting in decreased neuroinflammation and neuronal mortality. Furthermore, by limiting the inflammatory response and enhancing neuronal survival, overexpression of TRIM67 dramatically decreased cerebral ischemia‒reperfusion injury in MCAO/R-challenged mice and OGD/R-stimulated cells. Therefore, maintaining TRIM67 expression might constitute a promising therapeutic target for the treatment of ischemic stroke.

TRIM proteins play a crucial role in the regulation of NF-κB-dependent inflammation [20]. According to studies from several research groups, TRIM proteins regulate NF-κB pathways by ubiquitinating NF-κB-associated transcription factors, kinase proteins, and adaptor proteins [20]. Some TRIM proteins, such as TRIM30 and TRIM38, suppress NF-κB signaling by targeting the kinase proteins TAB2/3 and the IKK complex [21, 22]. Some TRIM proteins, however, including TRIM4, TRIM25, and TRIM45, operate as positive regulators of NF-κB pathways [30, 39, 40]. They specifically target RIG-I or TAB2 for K63-linked polyubiquitination, promoting RIG-I or TAB2-mediated NF-κB activation. More notably, prior research revealed that TRIM67 was markedly decreased after ischemic stroke [27] and could inhibit NF-κB activation and lessen the release of inflammatory components, hence having a certain anti-inflammatory impact [26]. The probable mechanism of TRIM67 was investigated by specifically targeting IκBα, a crucial negative regulator of NF-κB signaling.

In our work, we further revealed that TRIM67 is a neuroprotective molecule against cerebral ischemia‒reperfusion injury. We discovered that TRIM67 conjugates the K63-linked polyubiquitin chain to IκBα but not the K48-linked polyubiquitin chain, increasing its stability and leading to the inhibition of NF-κB signaling. These results are consistent with earlier studies; K48-linked polyubiquitin chains primarily target proteins for degradation, while K63-linked polyubiquitin chains seldom result in substrate degradation. Instead, K63-linked polyubiquitin chains make it easier for other proteins with specific ubiquitin-binding domains to attach with low affinity. TRIM67 overexpression decreased brain infarct size and neurological impairment and improved cognitive performance in MCAO/R mice, all of which have potential translational implications. Because AAV-mediated gene overexpression takes a certain amount of time in vivo, we pre-treated mice with AAV stereotactical injections 4 weeks before MCAO operation in this study, thus more detailed work will be needed to verify the therapeutic effect with a post treatment. On the other hand, systemically administration with an agonist which could selectively upregulate TRIM67 enzyme activity or enhance its interaction with IκBα may provide protective effects against cerebral ischemia–reperfusion injury. Certainly, these hypotheses remain to be determined in future studies. In addition, we examined the role of TRIM67 in cerebral ischemia‒reperfusion injury by extensive overexpression but did not specify which cells were involved. Therefore, more research is needed to determine in which cells it functions.

TRIM67, as a type of E3 ubiquitin‑linked enzyme, is involved in the posttranscriptional modification of certain proteins [41]. The ubiquitin conjugation system's specificity stems from the E3 ligase’s direct interaction with its substrates [42, 43]. Here, our findings indicated that TRIM67 interacted with IκBα, triggering its K63-linked ubiquitination. Therefore, more research is needed to clarify whether TRIM67’s E3 ligase activity is required for its function and which domain of TRIM67 is required for its interaction with IκBα.

The two main pathophysiologies of cerebral ischemia‒reperfusion injury, inflammation and apoptosis, are closely related. Numerous investigations have shown that inflammatory substances may not only directly cause nerve injury [44, 45] but also contribute to neuronal apoptosis [46, 47], thereby exacerbating cerebral ischemia‒reperfusion injury. As a result, inflammation is crucial in the progression of ischemic stroke. A previous study demonstrated that TRIM67 could significantly reduce NF-κB activation [26], a critical inflammatory regulatory pathway, and the level of inflammatory factors (IL-1β, IL-6, TNF-α). Studies have indicated that the excessive release of pro-inflammatory factors could lead to neuronal apoptosis dependent on caspase-3 pathway activation, and the application of zDVED-FMK (a caspase-3-specific inhibitor) can decrease neuronal apoptosis induced by cerebral ischemia‒reperfusion injury [48–50]. In our study, the results demonstrated that the number of TUNEL-positive cells, Bax, cleaved caspase-3, cleaved caspase-9, PARP, IL-1β, IL-6 and TNF-α protein expression were enhanced after cerebral ischemia‒reperfusion injury, while antiapoptotic Bcl-2 protein expression was decreased. Furthermore, IL-1β, IL-6 and TNF-α mRNA levels were enhanced after cerebral ischemia‒reperfusion injury, consistent with previous reports. Conversely, TRIM67 upregulation significantly suppressed the levels of Bax, cleaved caspase-3, cleaved caspase-9, and PARP and elevated the levels of Bcl-2, reducing the number of TUNEL-positive cells and the levels of inflammatory factors (IL-1β, IL-6, TNF-α) in vivo. These findings showed that by preventing the production of proinflammatory molecules, TRIM67 might reduce inflammation and apoptosis.

## Conclusions

In summary, we demonstrated that TRIM67, an E3 ligase, protects against inflammation and apoptosis by inhibiting NF-κB signaling pathways. TRIM67 interacts with IκBα and potentiates its K63-linked ubiquitination but reduces its K48-linked ubiquitination, enhancing the stability of the IκBα protein. Finally, our research identifies TRIM67 as a potential therapeutic target. Overall, our findings add to the growing body of knowledge on the role of TRIM family members in the treatment of cerebral ischemia‒reperfusion injury.

## Supplementary Information

**Additional file 1: Figure S1.** The expression of TRIM67 in primary cultured neurons decreased after OGD/R treatment.RT-qPCR tests demonstrating the level of Trim67 mRNA in primary cultured neuron exposed to OGD/R.Western blotting demonstrating level of TRIM67 protein expression in primary cultured neuron exposed to OGD/R.Analysis of TRIM67 expression quantified and compared to that of β-actin. The mean ± SD are displayed for all data. **p < 0.01, ***p < 0.001.

**Additional file 2: Figure S2.** The expression of TRIM67 in primary cultured microglia decreased after OGD/R treatment.RT-qPCR tests demonstrating the level of Trim67 mRNA in primary cultured microglia exposed to OGD/R.Western blotting demonstrating level of TRIM67 protein expression in primary cultured microglia exposed to OGD/R.Analysis of TRIM67 expression quantified and compared to that of β-actin. The mean ± SD are displayed for all data. *** *p* < 0.001.

**Additional file 3: Figure S3.** AAV-Vector or AAV-TRIM67 were stereotactically injected into hippocampus CA1 region, cerebral cortex and striatum of mice. After four weeks, the total mRNA or proteins of hippocampus CA1 region, cerebral cortex and striatum were harvested.RT-qPCR was conducted to examine Trim67 mRNA level.Western blotting was conducted to examine the protein level of TRIM67.The data were statistically analyzed in. n = 3. Values were displayed as mean ± SD. ***p < 0.001, ****p < 0.0001.

**Additional file 4: Table S1.** Antibodies employed in this study.

**Additional file 5: Table S2.** Primers used in this study.

## Data Availability

The authors declare that all data supporting the findings of this study are available within the paper and its Additional information files.

## Abbreviations

AAV: Adeno-associated virus
ACA: Anterior cerebral artery
Ad: Adenoviruses
BSA: Bovine serum albumin
CBF: Cerebral blood flow
CCA: Common carotid artery
Co-IP: Co-immunoprecipitation
CNS: Central nervous system
DMEM: Dulbecco's modified Eagle's medium
ECA: External carotid artery
ELISA: Enzyme-linked immunosorbent assay
FBS: Fetal bovine serum
ICA: Internal carotid artery
IKK: IκB kinase
MCAO/R: Middle cerebral artery occlusion and reperfusion
mNSS: Modified neurological severity score
MWM: Morris water maze
NF-κB: Nuclear factor kappa B
NOR: Novel object recognition
OGD/R: Oxygen–glucose deprivation and reperfusion
RIPA: Radioimmune-precipitation assay
RT-qPCR: Real-time quantitative PCR
SPF: Specific pathogen-free
TRIM67: Tripartite motif-containing 67
TUNEL: TdT-mediated dUTP-X nick end labeling
TTC: 2,3,5-Triphenyltetrazolium chloride
