# Supplementary figures and images for: TRIM67 alleviates cerebral ischemia‒reperfusion injury by protecting neurons and inhibiting neuroinflammation via targeting IκBα for K63-linked polyubiquitination
Source: Cell Biosci. 2023 May 29;13:99. doi: 10.1186/s13578-023-01056-w (PMC10226213; doi:10.1186/s13578-023-01056-w)

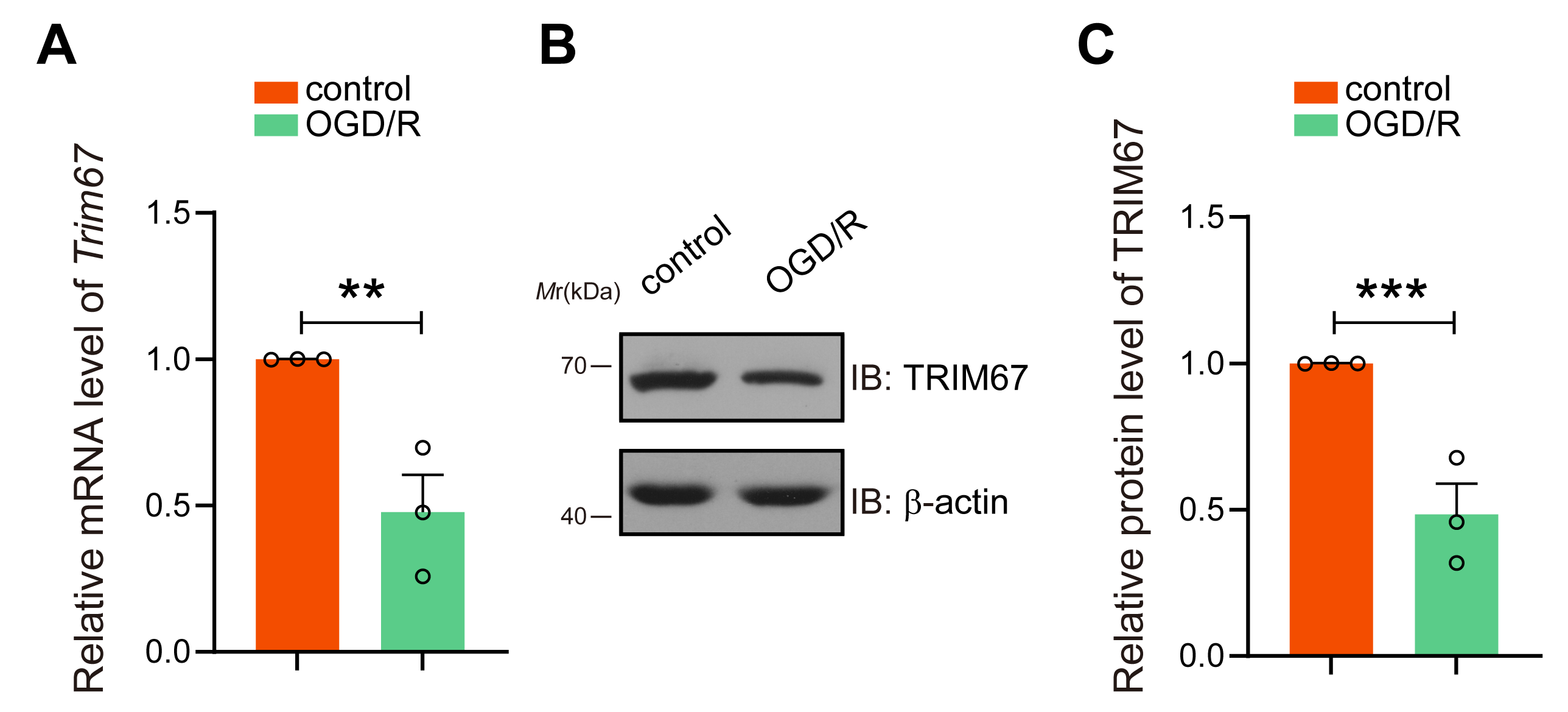

Supplement: Supplementary file 1 — Additional file 1: Figure S1. The expression of TRIM67 in primary cultured neurons decreased after OGD/R treatment.RT-qPCR tests demonstrating the level of Trim67 mRNA in primary cultured neuron exposed to OGD/R.Western blotting demonstrating level of TRIM67 protein expression in primary cultured neuron exposed to OGD/R.Analysis of TRIM67 expression quantified and compared to that of β-actin. The mean ± SD are displayed for all data. **p < 0.01, ***p < 0.001. [file 13578_2023_1056_MOESM1_ESM.tif]

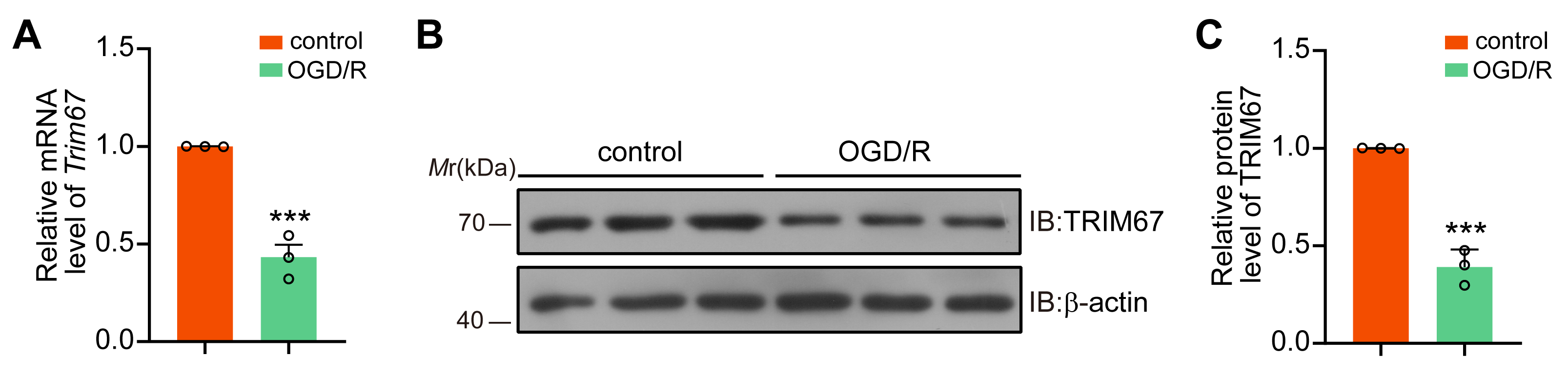

Supplement: Supplementary file 2 — Additional file 2: Figure S2. The expression of TRIM67 in primary cultured microglia decreased after OGD/R treatment.RT-qPCR tests demonstrating the level of Trim67 mRNA in primary cultured microglia exposed to OGD/R.Western blotting demonstrating level of TRIM67 protein expression in primary cultured microglia exposed to OGD/R.Analysis of TRIM67 expression quantified and compared to that of β-actin. The mean ± SD are displayed for all data. *** p < 0.001. [file 13578_2023_1056_MOESM2_ESM.tif]

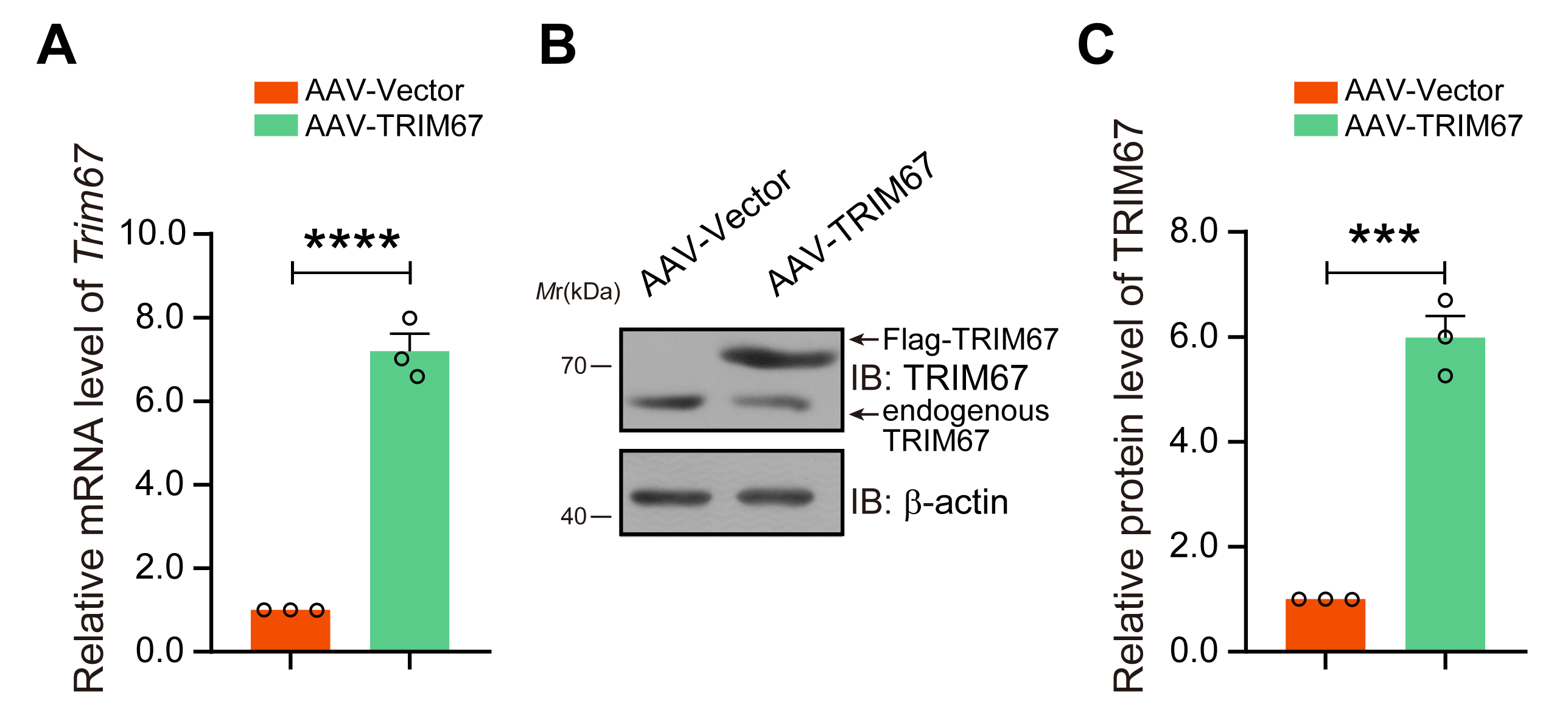

Supplement: Supplementary file 3 — Additional file 3: Figure S3. AAV-Vector or AAV-TRIM67 were stereotactically injected into hippocampus CA1 region, cerebral cortex and striatum of mice. After four weeks, the total mRNA or proteins of hippocampus CA1 region, cerebral cortex and striatum were harvested.RT-qPCR was conducted to examine Trim67 mRNA level.Western blotting was conducted to examine the protein level of TRIM67.The data were statistically analyzed in. n = 3. Values were displayed as mean ± SD. ***p < 0.001, ****p < 0.0001. [file 13578_2023_1056_MOESM3_ESM.tif]
