# Supplementary material for: TRIM67 alleviates cerebral ischemia‒reperfusion injury by protecting neurons and inhibiting neuroinflammation via targeting IκBα for K63-linked polyubiquitination
Source: Cell Biosci. 2023 May 29;13:99. doi: 10.1186/s13578-023-01056-w (PMC10226213; doi:10.1186/s13578-023-01056-w)
Supplement: Supplementary file 4 — Additional file 4: Table S1. Antibodies employed in this study. [file 13578_2023_1056_MOESM4_ESM.docx]

**Table S1. Antibodies employed in this study.**

| Antibody | Species | Type | IB | IF | Source | Identifier |
| --- | --- | --- | --- | --- | --- | --- |
| HA | Mouse | Mono- | 1:1000 |  | Santa Cruz | sc-7392 |
| Flag | Mouse | Mono- | 1:2000 |  | Santa Cruz | sc-166355 |
| Myc | Mouse | Mono- | 1:1000 |  | Santa Cruz | sc-40 |
| TRIM67 | Rabbit | Poly- | 1:1000 | 1:200 | Proteintech | 24369-1-AP |
| β-actin | Mouse | Mono- | 1:1000 |  | Santa Cruz | sc-47778 |
| NF-κB p65 | Rabbit | Mono- | 1:1000 | 1:200 | Cell Signaling | #8242 |
| Phospho-NF-κB p65 | Rabbit | Mono- | 1:1000 |  | Cell Signaling | #3033 |
| Phospho-IKKβ | Rabbit | Mono- | 1:1000 |  | Cell Signaling | #8943 |
| Phospho-IKKα/β | Rabbit | Mono- | 1:1000 |  | Cell Signaling | #2697 |
| IκBα | Mouse | Mono- | 1:1000 |  | Cell Signaling | #4814 |
| Phospho-IκBα | Rabbit | Mono- | 1:1000 |  | Cell Signaling | #2859 |
| α-tubulin | Mouse | Mono- | 1:2000 |  | Santa Cruz | sc-8035 |
| Histone H3 | Rabbit | Mono- | 1:2000 |  | Cell Signaling | #4499 |
| Bcl-xL | Rabbit | Mono- | 1:1000 |  | Cell Signaling | #2764 |
| Bax | Rabbit | Mono- | 1:1000 |  | Cell Signaling | #41162 |
| cleaved caspase-3 | Rabbit | Mono- | 1:1000 |  | Cell Signaling | #9664 |
| cleaved caspase-9 | Rabbit | Mono- | 1:1000 |  | Cell Signaling | #20750 |
| cleaved PARP | Rabbit | Mono- | 1:1000 |  | Cell Signaling | #5625 |

Abbreviations: IB, Immunoblotting; IF, Immunofluorescence.
