# Supplementary material for: TRIM67 alleviates cerebral ischemia‒reperfusion injury by protecting neurons and inhibiting neuroinflammation via targeting IκBα for K63-linked polyubiquitination
Source: Cell Biosci. 2023 May 29;13:99. doi: 10.1186/s13578-023-01056-w (PMC10226213; doi:10.1186/s13578-023-01056-w)
Supplement: Supplementary file 5 — Additional file 5: Table S2. Primers used in this study. [file 13578_2023_1056_MOESM5_ESM.docx]

**Table S2. Primers used in this study.**

| Primer name | Primer sequences (5’- 3’) | |
| --- | --- | --- |
|  | Forward | Reverse |
| Quantitative RT-PCR primers | | |
| *Trim45* | AAGATGTCAGAAATCAGGA | GCATCAGAGC GCCACGGTCC |
| *Il-1β* | GAAAGACGGCACACCCAC | TGTGACCCTGAGCGACCT |
| *Il-6* | TCTCTGGGAAATCGTGGAA | GATGGTCTTGGTCCTTAGCC |
| *Tnf-α* | ACGGCATGGATCTCAAAGAC | AGATAGCAAATCGGCTGACG |
| *Cxcl1* | GAGCTTGAAGGTGTTGCCCT | CGCGACCATTCTTGAGTGTG |
| *Ccl2* | GCAGGTCCCTGTCATGCTTC | GTGGGGCGTTAACTGCATCT |
| *β-actin* | CCTTCTTGGGTATGGAATCCTG | CAATGCCTGGGTACATGGTG |
